# Distributed Channel Ranking Scheduling Function for Dense Industrial 6TiSCH Networks

**DOI:** 10.3390/s21051593

**Published:** 2021-02-25

**Authors:** Ismael Amezcua Valdovinos, Patricia Elizabeth Figueroa Millán, Jesús Arturo Pérez-Díaz, Cesar Vargas-Rosales

**Affiliations:** 1 Facultad de Telemática, Universidad de Colima, PO 28040 Colima, Mexico; 2 División de Estudios de Posgrado e Investigación, Tecnológico Nacional de México Campus Colima, PO 28976 Colima, Mexico; patricia.figueroa@itcolima.edu.mx; 3 Tecnologico de Monterrey, Escuela de Ingeniería y Ciencias, PO 76130 Monterrey, Mexico; jesus.arturo.perez@tec.mx (J.A.P.-D.); cvargas@tec.mx (C.V.-R.)

**Keywords:** 6TiSCH, scheduling function, IEEE802.15.4e TSCH

## Abstract

The Industrial Internet of Things (IIoT) is considered a key enabler for Industry 4.0. Modern wireless industrial protocols such as the IEEE 802.15.4e Time-Slotted Channel Hopping (TSCH) deliver high reliability to fulfill the requirements in IIoT by following strict schedules computed in a Scheduling Function (SF) to avoid collisions and to provide determinism. The standard does not define how such schedules are built. The SF plays an essential role in 6TiSCH networks since it dictates when and where the nodes are communicating according to the application requirements, thus directly influencing the reliability of the network. Moreover, typical industrial environments consist of heavy machinery and complementary wireless communication systems that can create interference. Hence, we propose a distributed SF, namely the Channel Ranking Scheduling Function (CRSF), for IIoT networks supporting IPv6 over the IEEE 802.15.4e TSCH mode. CRSF computes the number of cells required for each node using a buffer-based bandwidth allocation mechanism with a Kalman filtering technique to avoid sudden allocation/deallocation of cells. CRSF also ranks channel quality using Exponential Weighted Moving Averages (EWMAs) based on the Received Signal Strength Indicator (RSSI), Background Noise (BN) level measurements, and the Packet Delivery Rate (PDR) metrics to select the best available channel to communicate. We compare the performance of CRSF with Orchestra and the Minimal Scheduling Function (MSF), in scenarios resembling industrial environmental characteristics. Performance is evaluated in terms of PDR, end-to-end latency, Radio Duty Cycle (RDC), and the elapsed time of first packet arrival. Results show that CRSF achieves high PDR and low RDC across all scenarios with periodic and burst traffic patterns at the cost of increased end-to-end latency. Moreover, CRSF delivers the first packet earlier than Orchestra and MSF in all scenarios. We conclude that CRSF is a viable option for IIoT networks with a large number of nodes and interference. The main contributions of our paper are threefold: (i) a bandwidth allocation mechanism that uses Kalman filtering techniques to effectively calculate the number of cells required for a given time, (ii) a channel ranking mechanism that combines metrics such as the PDR, RSSI, and BN to select channels with the best performance, and (iii) a new Key Performance Indicator (KPI) that measures the elapsed time from network formation until the first packet reception at the root.

## 1. Introduction

Industrial environments demand high reliability for safety-critical messages, low latency, often demanding real-time communication guarantees, resistance to background noise produced by large machinery, wireless network coexistence in the Industrial-Scientific-Medical (ISM) band, fault-tolerance to allow networks to continue functioning in case of node failure, link reliability to avoid high packet loss and thus high delays, and scalability [1]. Wired solutions serve these requirements at high costs of installation and maintenance [2]. To this end, several working groups have been developing a new breed of protocols to support wireless communications in harsh industrial environments such as WirelessHART, ZigBee, ISA100.11a, and WIA-PA. These technologies are supported by the IEEE 802.15.4 standard [3].

The Institute of Electrical and Electronics Engineers Standards Association (IEEE-SA) published the IEEE 802.15.4e amendment [4] in 2012 to enhance and extend the functionalities of the IEEE 802.15.4-2011 standard. These enhancements consist of several Medium Access Control (MAC) behaviors, such as the Deterministic and Synchronous Multichannel Extension (DMSE) that targets applications with stringent Quality of Service (QoS) requirements such as deterministic latency, high reliability, and scalability; the TSCH, which provides high reliability and time-critical assurances; the Low Latency Deterministic Network (LLDN) targeting applications that typically demand robustness; RFID-based IEEE 802.15.4e; and the asynchronous multi-channel adaptation, which uses the non-beacon enabled mode of the IEEE 802.15.4e amendment [5].

In the TSCH mode of the IEEE 802.15.4e amendment, nodes communicate by following a Time Division Multiple Access (TDMA) schedule combined with frequency hopping, which improves network reliability by mitigating the effects of interference and multi-path fading. Moreover, the purpose of the IEEE 802.15.4e document is to define link-layer mechanisms for communication. The specification does not define how the communication schedule is built and matched to the traffic requirements of the network [6]. The IPv6 over the TSCH mode of the IEEE 802.15.4e standard Working Group (6TiSCH WP) defines a sublayer that allows a scheduling policy to manage TSCH schedules in the network [7].

The scheduling policy, referred to as the Scheduling Function (SF) from now on, plays an important role in 6TiSCH networks since it dictates when (timeslot) and where (channel offset) the nodes are communicating according to the specific requirements of the application. Therefore, the SF is responsible for the allocation, relocation, and deallocation of cells based on the application requirements. Efficient schedules are directly related to the performance on metrics such as the end-to-end latency, PDR, and Radio Duty Cycle (RDC) of a network. As mentioned before, complex industrial processes often require strict control mechanisms and scalable diagnostic transport. Industrial networks rely on technologies that can provide ultra-high reliability while operating in harsh environments. Communication failures in such networks can lead to catastrophic consequences [7]. Moreover, typical industrial environments consist of heavy machinery and complementary wireless communication systems that can create interference [8,9]. Therefore, it is crucial to define an efficient and robust SF that can overcome the challenges present in harsh industrial environments.

A robust SF for 6TiSCH industrial networks must define efficient bandwidth estimation and channel selection mechanisms. Current approaches in Scheduling Functions (SFs) provide efficient bandwidth estimation mechanisms with weak random or sequential channel selection. Another set of related work focuses only on efficient channel selection without defining bandwidth estimation mechanisms. Section 3 describes the gap between related work. As mentioned before, industrial environments often experience high interference due to heavy machinery or complementary wireless communication systems. Hence, it is important to define an SF that provides efficient channel selection based on several metrics such as PDR, RSSI, and Background Noise (BN); and robust bandwidth estimation mechanisms that can adapt dynamically to different traffic patterns and topologies.

The intention of this paper is to present a new scheduling mechanism that effectively builds distributed TSCH schedules using 6TiSCH and IEEE 802.15.4e networks by defining efficient bandwidth estimation and channel selection mechanisms. The Channel Ranking Scheduling Function (CRSF) uses several metrics to ensure the selection of the highest quality channels even under heavy interference. We define a high-quality channel as one with high PDR, strong RSSI, and low BN. Moreover, the bandwidth estimation/allocation mechanism uses a one-dimensional Kalman filtering technique to avoid over-provisioning of cells when burst traffic patterns are present in the network. The contributions of the paper are the following:

- It provides a buffer-based bandwidth allocation mechanism that uses Kalman filtering to effectively determine the number of timeslots required at a given time.
- It provides a channel ranking mechanism that uses PDR statistics, RSSI, and the BN metrics to efficiently rank channels.
- It proposes a new KPI that measures the elapsed time from network formation until first packet reception at the root or sink node.

The rest of the paper is organized as follows: An overview of the IEEE 802.15.4e standard and the use of IPv6 over the TSCH mode of such networks is covered in Section 2. Section 3 provides an in-depth analysis of proposed scheduling policies and mechanisms. Section 4 defines our CRSF as an alternative to build TSCH schedules in 6TiSCH networks. Section 5 depicts the experimental setup used to evaluate performance of our scheduling and function. Section 6 provides an analysis of our findings. Finally, Section 7 draws final remarks, conclusions, and future work.

## 2. Technical Background

This section briefly describes the TSCH mechanisms defined by the IEEE 802.15.4e amendment. It also describes the mechanisms defined by the 6TiSCH working group that allow building schedules in TSCH networks, prior to introducing scheduling policies and approaches in the next section.

### 2.1. The IEEE 802.15.4e TSCH Mode

**Figure 1:**
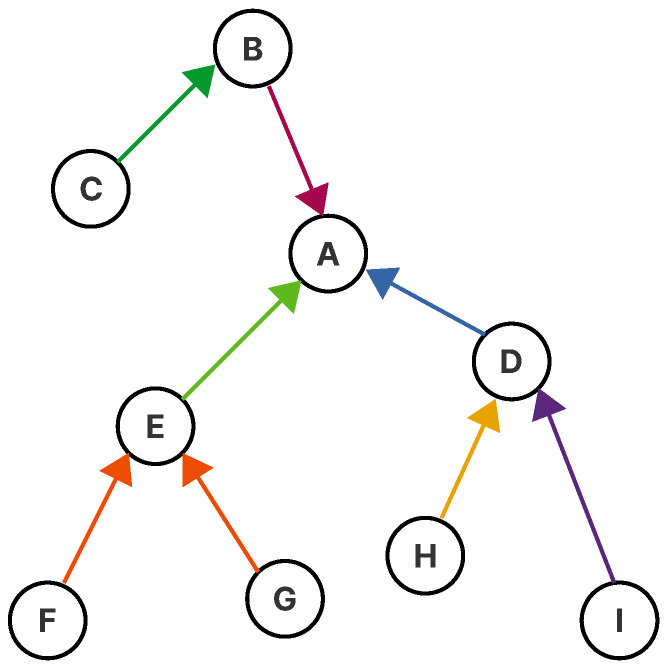
The Routing Protocol for Low-Power and Lossy Networks (RPL) constructs a tree-like routing topology, called the Destination-Oriented Directed Acyclic Graph (DODAG), rooted at one or more border router. Each node has a rank that defines the routing distance from said node to its root.

TSCH combines time-slotted access and channel hopping to provide large network capacity, high reliability, and predictable latency. It can be used with any network topology, but is particularly well-suited for multi-hop networks where multi-channel communication allows for an efficient use of available resources [10]. Figure 1 shows how the IPv6 Routing Protocol for Low-Power and Lossy Networks (RPL) organizes an existing topology into a multi-hop routing structure for upward traffic, ranking each node based on its proximity to the root or sink node (Node A in Figure 1) in the network. In TSCH, a parent node is called the time-source node or neighbor because it uses its time to downwardly synchronize clocks.

**Figure 2:**
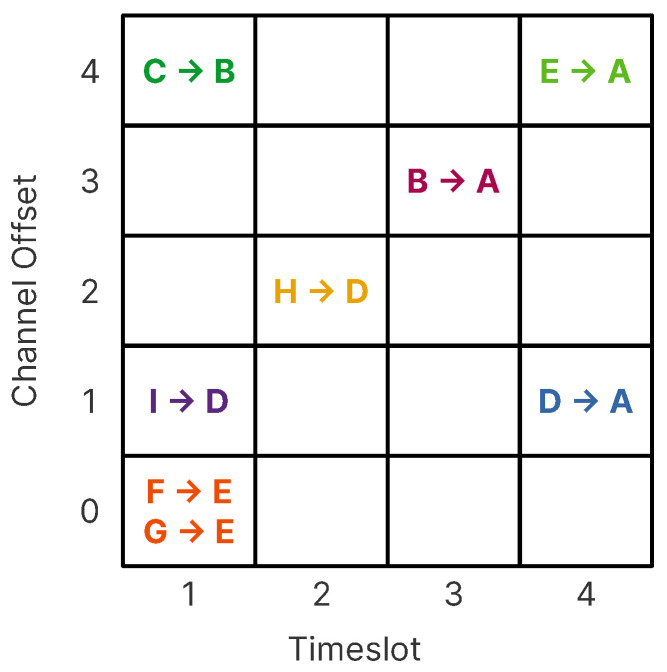
Example of how a many-to-one schedule on a five-channel deployment can be constructed using the RPL topology defined in Figure 1. The channel offset translates to the radio frequency used for the transmission of the frame, and the timeslot is the time window assigned to each node to send information to the sink.

Nodes synchronize on a periodic slotframe (In a TSCH network, the concept of the superframe used in IEEE 802.15.4-2012 is replaced with a slotframe. The latter contains defined periods of communication between peers that may be either CSMA-CA or guaranteed, automatically repeating based on the node’s shared notion of time.), which consists of a fixed number of timeslots (slotframe length). Each timeslot is used to send data frames and receive the related acknowledgment. Since all devices share common time and channel information, devices may hop over the entire channel space to minimize the negative effects of multi-path fading and interference, whilst avoiding collisions and, therefore, the need for retransmissions. Both features are desirable for operation in harsh industrial environments [4]. Figure 2 shows how a schedule can be constructed based on the RPL structure and topology of a network. The building of such a schedule is not part of the standard, and it is left open for implementers to search for suitable mechanisms for its creation.

On the multichannel aspect, TSCH has 16 different channels available for communication. Each channel is identified by its channel offset, i.e., an integer ranging from zero to 15. As mentioned earlier, the IEEE 802.15.4 radio technology uses ISM frequencies and therefore is highly susceptible to interference from appliances and other wireless networks. As we describe in Section 3, most TSCH deployments use allowlisting/blacklisting techniques to avoid channels that behave poorly in order to improve overall performance.

A link is an important concept in a TSCH network. It can be defined as the pair of the timeslot and channel offset used by two nodes in their schedule. Each channel can make use of either a dedicated or a shared link. Dedicated links are allocated to a single (sender/receiver) pair, handling deterministic traffic, periodic transmissions, and direct access to the channel. On the other hand, shared links can be used to exchange routing/scheduling information, to provide basic connectivity to nodes when dedicated links are not available using CSMA/CA and to add flexibility to the network. According to [4], a link can be established using the following equation:

(1) $$f=F\left[\left(\mathrm{channel}\;\mathrm{offset}+ASN)\;\mathrm{mod}\;\mathrm{length}\right)\right]$$

where *f* is the communication frequency that will be used to send the packet, ASN is the absolute number slot, *F* is a collection of possible channels that can be used for communication between both nodes (a function for the conversion to a frequency used by the transceiver), channeloffset is the number of channels that can be used (not all 16 channels are mandatory to use in an IEEE 802.15.4e network), and length is the length of *F* used to select unique channel hopping sequences.

To form a TSCH network, a coordinator advertises the presence of the network by sending Enhanced Beacons (EBs) with the following content: time information so new devices can synchronize, channel hopping information, timeslot information describing when to expect a frame to be transmitted, and initial link and slotframe information so new devices know when to listen for transmissions from the advertising device and when they can transmit. The joining device will typically go through a procedure to allocate additional communication resources (slotframes and links). The amount of slotframes and links required by the device is determined by a higher layer standard.

### 2.2. IPv6 over IEEE 802.15.4e TSCH

In 2007, the 6LoWPAN working group started working on the specifications for transmitting IPv6 packets over IEEE 802.15.4 networks [11] by defining an adaptation layer to compress IPv6 headers designed to fit the default IPv6 MTU size (1280 bytes) into a single IEEE 802.15.4 frame (127 bytes) [12]. Furthermore, the working group also focused on the auto-configuration of IPv6 addresses, the support of link-layer subnet broadcasting in shared networks, the reducing of routing and management overhead [13], the adoption of lightweight application protocols [14], and the support for security mechanisms (confidentiality and integrity protection, device bootstrapping, key establishment, and management).

The IEEE 802.15.4e amendment was published in 2012 defining link-layer mechanisms to support a TSCH scheme that seeks to alleviate multipath fading and interference problems present in dense industrial wireless networks. To enable the convergence of Internet protocols in such networks, the Internet Engineering Task Force (IETF) created a working group called 6TiSCH (IPv6 over IEEE 802.15.4e TSCH mode of IEEE 802.15.4e), which defines a 6topsublayer that provides the abstraction of an IP link over a TSCH MAC and schedules packets over TSCH cells [15].

6TiSCH aims to link IEEE 802.15.4e TSCH’s capabilities with prior IPv6-enabled standards such as IETF 6LoWPAN, RPL, and the Constrained Application Protocol (CoAP). 6TiSCH inherits the capability of performing centralized route computation to achieve deterministic properties, but also adds capabilities for distributed routing and scheduling operations based on the RPL protocol.

### 2.3. Schedule Management in 6TiSCH Networks

Scheduling in TSCH involves a Scheduling Function (SF) that can make use of several mechanisms to manage the schedule. Such a policy is in charge of determining which timeslot is allocated to which nodes, and it is not standardized by the 6TiSCH WG in order to provide flexibility in requirements for constrained deployments [7].

One alternative to scheduling in 6TiSCH networks is distributed scheduling, in which neighbor nodes negotiate which timeslots to use with one another. 6TiSCH provides mechanisms to exchange these messages by using 6P Transactions (The 6top Protocol (6P) allows two neighbor nodes to update their TSCH schedules. A 6P Transaction is the complete negotiation between these two nodes and is issued by an SF in order to dynamically add, delete, or reallocate cells into their schedules [16]) to allocate/relocate cells dynamically.

## 3. Related Work

Scheduling in a TSCH network involves policies that help to determine timeslots to be allocated to which nodes by making use of several mechanisms and link quality-related parameters. As discussed earlier, such a scheduling policy is not standardized by the 6TiSCH working group and is left open for researchers and developers to provide a policy that fits their needs. For a detailed taxonomy on scheduling policies for 6TiSCH, please refer to [17,18].

### 3.1. Centralized Scheduling

Palattella et al. [19] introduced the Traffic-Aware Scheduling Algorithm (TASA), a centralized algorithm that requires complete network topology information to build schedules based on the amount of static traffic load generated by each node in the network.

Farías and Dujovne [20] proposed a Path Computation Element (PCE)-enabled scheduling approach in which the number of allocated slots is chosen according to the overload value of the link (the number of descendants that share the available bandwidth of the link).

Centralized scheduling mechanisms provide high reliability schedules since they optimize links based on information about the network topology and traffic patterns. Moreover, centralized scheduling is used when the network topology does not vary over time and the traffic pattern is well known. Therefore, it is hard for this kind of scheduling to support varying traffic patterns such as burst traffic commonly used in industrial and home networks with dynamic node deployments.

### 3.2. Distributed Scheduling

As a starting point, the 6TiSCH WP defined a minimal scheduling function called Scheduling Function Zero (SF0) [21], which uses a Cell Estimation Algorithm (CEA) to calculate the number of cells required for communication plus over-provision cells to reduce the probability of packet loss in case more cells are required. Channel selection in SF0 is performed randomly. An Allocation Policy (AP) module uses Packet Delivery Ratio (PDR) statistics to monitor currently allocated cells for relocation in the case when PDR measurements are below a certain threshold. Chang et al. [22] showed that SF0 accordingly allocates/relocates cells when bandwidth requirements from devices change. The scheduling function was evaluated using the OpenWSN sensor network simulator. The use of PDR solely as a performance metric requires the exchange of a considerable number of packets to be transmitted in order to compute statistically valid values. Moreover, random cell selection often leads to poor channel selection due to interference or low RSSI.

Domingo-Prieto et al. [23] proposed a distributed scheduling policy based on the Proportional, Integral, and Derivative (PID) control algorithm. A PID is a feedback mechanism used for the stabilization of industrial control loops where decisions are performed according to the current state and a desired end state of the system (in this case, approach a packet queue size close to zero). It uses the size of the queue and the number of unused scheduled slots in a slotframe as inputs. PID scheduling avoids constant cell relocation due to its feedback mechanism. Moreover, it also uses random cell selection, which is not optimal in scenarios where heavy interference from multiple sources is present.

Muraoka et al. [24] introduced the notion of housekeeping and random scheduling to provide collision-free schedules. The authors provided two housekeeping approaches: Tx-housekeeping runs on the transmitting node to detect schedule collisions by comparing performance on different cells aimed at the same neighbor, i.e., when the PDR of that cell is significantly lower than the other cells in the bundle; and RX-housekeeping runs on the receiving node to detect collisions by overhearing packets on the receiving node, which is not the neighbor it expects packets from, i.e., if a scheduled cell on the overhearing node is used by other neighbors while idle, it relocates that cell to prevent future collisions. When using large slotframe lengths, such as 101, random cell allocation has a low probability of choosing already allocated cells. However, as node deployment increases and the slotframe length decreases, i.e., 17 timeslots per slotframe, the probability of randomly choosing allocated cells also increases. Moreover, channel quality is not taken into account for random cell selection.

Accettura et al. [25] proposed the Decentralized Traffic Aware Scheduling (DeTAS), an algorithm based on TASA for building optimum collision-free schedules in multihop IEEE 802.15.4e networks. In DeTAS, all devices are assumed to be synchronized within the same slotframe. Each node needs to know the amount of traffic it will receive from its children and the amount of traffic it will send to the parent. It uses neighbor-to-neighbor signaling in order to gather network and traffic information. DeTAS introduces a slight overhead by defining signaling MAC frames.

Aijaz and Raza [26] introduced, a Decentralized Adaptive Multihop Scheduling Protocol (DeAMON) that builds its schedule based on the traffic generated and received by a node. Each node selects its own timeslot and channel offset based on the following rules: (1) any node starts a scheduling process if it is a leaf node; (2) a node selects the minimum possible timeslots on the available channel offset where there are no conflicts as per its local knowledge; (3) any parent node confirms the scheduling if there are no conflicts as per its local knowledge; (4) any node upon overhearing scheduling messages from siblings updates its local knowledge and forwards this information to its parent; and (5) any parent confirms request allocations based on said rules and its local knowledge. DeAMON allocates cells in a sequential manner to each node in the network and does not consider link quality-related constraints, which may incur a low PDR when using low quality or highly interfered channels.

Chang et al. [27] proposed the Low Latency Scheduling Function (LLSF) algorithm. At the first hop, LLSF selects a random cell among the unscheduled timeslots in the slotframe. For each reception from the previous hop, it determines the number of slots between the current and the previous slots from the same neighbor based on the slotframe size. It selects the slot with the largest gap to the left. The new transmission slot to the next hop is the closest unused slot to the right of the selected reception slot. According to the authors, when using LLSF, end-to-end latency is reduced up to 82.8% compared to the latency when using SF0 [21].

Karaagac et al. [28] used the joint coordination and interaction of distributed and centralized scheduling mechanisms for 6TiSCH networks that allow the coexistence of time-sensitive and scalable IIoT applications. The design and implementation of such mechanisms are based on the CoAP Management Interface (CoMI) [29]. There are two logical entities that can define schedules: the local scheduling entity, used to monitor the state of the network and to determine schedules based on local network views; and the centralized entity, which collects the network state, topology, and current schedules to calculate routes and schedules based on the global view of the network. Due to the large number of signaling messages, a cost analysis is also provided where the amount of data and channel resources required to be exchanged is discussed. Experimental analysis shows that hybrid scheduling mechanisms provide acceptable performance with centralized scheduling, although deterministic flows with dedicated paths, reserved resources, and heavy signaling are still crucial in order to provide deterministic behavior.

In order to provide a model to determine the number of cells to be scheduled, Palattella et al. [30] described the On-The-fly (OTF) algorithm. The latter monitors the amount of data being sent to each of the mote’s neighbors. When the amount of data becomes too large compared to the number of cells scheduled for that neighbor, OTF asks 6top to add cells into that particular schedule. It uses a configurable threshold that needs to be optimized in order to avoid the unnecessary allocation/relocation of cells. OTF does not provide a scheduling function; instead, it delegates the task to the 6top sublayer. Thus, effective cell allocation may not be achieved under heavy channel interference present in industrial environments. More recently, Righetti et al. [31] provided enhancements to OTF, namely the Enhanced OTF (E-OTF), by introducing a mechanism to recover from network congestion.

Duquennoy et al. [32] introduced Orchestra, a distributed approach to scheduling in TSCH+RPL networks. In Orchestra, nodes adapt their schedule by exploiting information from the RPL topology and following a set of deployment-specific scheduling rules. The key idea is to provide a single slot for each neighbor, defined in a way that can be installed/removed if the RPL topology changes. Orchestra is not collision free and not good for burst applications as it uses single cell allocation using the same channel and does not dynamically resolve slot conflicts [33].

The Minimal Scheduling Function (MSF) [34] uses autonomous and negotiated cell allocation, and it is optimized for its use in upstream traffic. Autonomous cells are computed as a hash of the 64-bit Extended Unique Identifier (EUI64) address of the node for Rx and as a hash of the Layer 2 EUI64 address for Tx. If a node determines that the number of link-layer frames required to send to its parent is larger than the negotiated cells, MSF triggers a 6P Transaction to schedule negotiated cells. MSF schedules one cell at a time. Hauweele et al. [35] argued that MSF is subject to over-provisioning cells, especially in the case of varying traffic load.

Hamza and Kaddoum [36] provided an enhancement to the bandwidth allocation algorithm defined in MSF, called E-MSF, by computing the mean of packets generated by each node and using a Poisson-based prediction model for possible required cells in the next slotframe. Results show that E-MSF outperforms MSF in terms of end-to-end latency, because it constantly keeps probable cells scheduled in the slotframe, whether they are being used or not. This may incur cell starvation if the slotframe length is small and the number of nodes in the network is high.

### 3.3. Effective Channel Selection

One limitation of the IEEE 802.15.4e TSCH mode is that all 16 channels are used indiscriminately, even when channels are experiencing different levels of interference. Blacklisting mechanisms are often used to alleviate channel inefficiency based on statistical measurements, such as PDR. Such approaches have disadvantages: if the number of packets sent is too small (low statistical significance), the resulting PDR measurement may be unsteady; on the other hand, if the number of packets sent is too large, the resulting PDR measurement may be resistant to dynamical spectral condition changes.

An early work on channel modeling was presented by Watteyne et al. [37], where the authors evaluated the benefits of path and frequency diversity with particular focus on the impact on network routing. The authors stated that interference and persistent multipath fading cause the PDR of links to vary with the channel. By simulating the performance of a single channel and channel hopping solutions on traces from a real-world WSN deployment, the authors showed that even blind channel hopping improves connectivity and reduces the expected transmission count (i.e., the number of packets needed to be transmitted before successful arrival, $ETX=\frac{1}{PDR}$) by 56%, stating that performance can be further improved by allowlisting channels on a link-by-link basis. The Expected Transmission Count (ETX) is considered as a good metric for WSN performance since packet retransmissions are a major source of energy and time expenditure.

Li et al. [38] proposed an adaptive channel selection scheme based on the multi-arm bandit problem (In the multi-arm bandit problem, there are *N* independent arms, and one arm can be selected and pulled each time unit with two outcomes: success and failure. The target is to maximize the expected times of success by deciding which arm to select each time.). Each channel is considered as one independent slot machine, the inputs of which are weighted historical successful transmissions, historical failed transmissions, and the disabled channels. It uses a window-based mechanism to detect the dynamics of the environment, which reduces the probability of sudden index value changes. Results show that such an adaptive mechanism improves throughput when compared to random channel hopping schemes.

Gomes et al. [39] also modeled channel selection as a multi-armed bandit optimization problem. The authors argued that blacklisting frequencies alone are not sufficient due to the spatial-temporal variations of channel quality. The Multihop and Blacklist-based Optimized TSCH protocol (MABO-TSCH) uses a distributed blacklist to improve performance in multihop wireless networks with high levels of external interference and multi-path fading. Results show that MABO-TSCH is effective in both periodic and non-periodic data traffic models. However, it requires large intervals to converge and heavy signaling (blacklisting information is piggybacked in data packets) to outperform an ideal centralized solution (local blacklisting).

Du and Roussos proposed A-TSCH [40], an Adaptive mechanism for channel selection in TSCH networks. A-TSCH introduces an ancillary Noise Floor (NF) slot type to the schedule. The algorithm regulates that no communication can take place in NF slots in order to sense each channel looking for background noise. Two slots are placed at the rear of every IEEE 802.15.4e superframe. The noise floors are collected by accessing the RSSI. In A-TSCH, it is crucial that nodes maintain knowledge of their neighbors’ blacklist so that senders and receivers use identical hopping sequences when communicating.

Tavakoli et al. [41] proposed ETSCH, an Enhanced version of the TSCH protocol that restricts channel usage to those measures providing good quality using non-intrusive channel quality estimations (passive probing). ETSCH incorporates three components into the legacy TSCH: a channel quality estimator called Non-Intrusive Channel quality Estimation (NICE), a channel hopping allowlisting algorithm, and an enhanced beacon hopping sequence list. ETSCH seeks channels that are less interfered by the coexistence of Wi-Fi (i.e., Channels 15, 20, 25, and 26) according to [37]. Results show that ETSCH achieves higher PDR than the legacy TSCH and A-TSCH while being non-intrusive by using silent periods of TSCH to measure channel quality.

Elsts et al. [42] investigated and compared different adaptive channel selection metrics. Two well-known channel quality assessment metrics were studied, namely the Packet Reception Rate (PRR) and the wireless medium noise levels during no transmission periods measured through periodic RSSI sampling. Results show that under heavy interference, using PRR as a channel quality assessment metric achieves close to a 100% PDR.

Finally, Tavakoli et al. [43] used the ETSCH with a Distributed Channel Sensing technique (ETSCH + DCS) to dynamically detect good quality channels to be used for communication. The premise is that non-coordinator nodes cannot determine silent periods due to synchronization loss caused by clock drifts, and therefore, it is not possible to use the NICE algorithm to compute channel quality assessment. The Clear Channel Assessment (CCA) packet is sued to extract information about the currently used channel each time a packet is received.

### 3.4. Related Work Summary

Current state-of-the-art works on the SF for 6TiSCH networks focus on either bandwidth allocation or effective channel selection, as can be seen in Table 1. Related work with sequential and random channel selection can lead to the use of low performing channels and thus low PDR measurements. Works related to enhancing TSCH with adaptive channels propose different metrics to assess the quality of the channels, but they do not provide bandwidth allocation mechanisms to estimate the number of required cells. In our work, the CRSF allows parameters such as the PDR, RSSI, and BN to select the best channel based on both statistical and channel performance metrics, covering the gap between bandwidth allocation and efficient channel selection.

## 4. Scheduling for Dense Industrial 6TiSCH Networks

This section discusses the two main components, namely the bandwidth allocation and channel selection processes, of our proposed CRSF for dense industrial networks. Effective channel selection and bandwidth allocation for scheduling in IEEE 802.15.4e TSCH networks for industrial deployments is of prime interest since such scenarios often involve heavy interference coming from multiple sources, e.g., Wi-Fi access points, coexisting WSN deployments, and heavy machinery. To build effective distributed schedules, nodes need to select the best routes for each link based on several metrics in order to provide acceptable rates of transmission.

### 4.1. Bandwidth Allocation

Bandwidth allocation in 6TiSCH networks refers to the problem of determining the number of slots required by a node for a given time to send and received data. The number of slots should be calculated based on the amount of traffic the node is sending to its parent. Optimal bandwidth allocation results in increased overall throughput.

We use a buffer-based bandwidth allocation scheme, where the sending node is constantly retrieving the number of packets in the buffer queue. If this number is greater than the current number of allocated slots, the algorithm will add more slots to its schedule. On the other hand, if the buffer queue size is lower than the current number of allocated links, the algorithm will remove links from its schedule.

As the number of packets in the buffer may change drastically, we propose the use of a Kalman filtering technique to prevent the constant addition and removal of links, which may result in extra signaling in the network. The Kalman filter minimizes the mean squared error between the measured and estimated data. In our case, the measurements are limited to the number of packets in the queue buffer. Therefore, a simplified one-dimensional Kalman filter is used. Algorithm 1 describes the process of determining the number of slots required to allocate/relocate to the TSCH schedule.
**Algorithm 1** Bandwidth allocation algorithm.    **Input** The current number of packetsInBuffer    **Output** The number of requiredCells    **while** Sending packets **do**     requiredCells←⌈Kalman(packetsInBuffer−allocatedSlots)⌉     **if**
requiredCells>allocatedSlots
**then**      addLinks(requiredCells)     **else if**
requiredCells<allocatedCells
**then**      removeOneLink()     **end if**    **end while**

We apply the Kalman filter to the required bandwidth parameter, which indicates how many cells are to be negotiated between nodes based on the amount of traffic in the sending node’s buffer. When the filter is applied to a one-dimensional variable, all matrices involved in the algorithm become also one-dimensional variables. Therefore, the cost of using a Kalman filter under such circumstances is minimal. As Algorithm 1 describes, the Kalman filter is used on sensor nodes to compare the current number of allocated cells with the required number of cells according to the buffer occupation.

### 4.2. Channel Selection

In order to provide effective channel selection and cell provisioning in 6TiSCH networks, we propose the CRSF. We focus on two main medium characteristics, namely the statistical performance of each link and the current environmental performance measured as passive probing. The scheduling function combines three metrics to accurately rank the best channels based on the current measurements of RSSI, BN, and PDR. Channels are ranked according to the following:

(2) $$CR_{i}=\sum_{i=0}^{N}\left[\ln\frac{1}{PDR_{i}}+\left(\ln\frac{1}{2RSSI_{i}}+\ln\frac{BN_{i}}{2}\right)\right]$$

where $CR_{i}$ is an unordered list of ranks based on statistical (PDR) and link-level performance (RSSI and BN) and *N* is the number of channels available in the TSCH deployment.

The function is composed of two main parts: the first part incorporates a statistical metric, namely the PDR, since it is a commonly used indicator for performance in WSNs due to its low computational complexity; the second part incorporates link-level metrics, namely the RSSI and BN, as indicators of the current performance of the wireless medium. This allows the CRSF to rank channels based on their network performance and the current characteristics of the physical medium. Furthermore, the function is modeled using the Exponential Weighted Moving Average (EWMA) filter, which allows adjusting the ratio at which each metric grows. From Equation (2), we can observe that the ratios are assigned as follows: 50% for PDR and 25% for both RSSI and BN.

As mentioned before, statistical metrics such as the PDR converge after network formation, since the number of packets at the beginning is low enough to give accurate estimations. Similarly, the RSSI and BN values in the Contiki-NG OS are calculated using EWMA filters. As this is a minimization function, the channels with lower values in the channel ranking CR are the best ranked channels at the moment. Therefore, we compute the reciprocal value of desirable values such as the PDR and RSSI (the more the PDR and RSSI, the lower the value is computed). Elsts et al. [42] investigated the wireless medium noise level parameter to assess quality on TSCH networks. Noise levels were measured through periodic RSSI sampling. The authors incorporated noise level metrics into the Contiki-NG OS to evaluate channel quality for possible reallocation of channels. For our BN parameter, we use the mechanisms already incorporated into Contiki-NG to model the background noise. The BN is a negative value in our system; therefore, we want to provide an increase in our computation of the ranking when high interference is present. We also use natural logarithms as a normalization function.

Channel rankings in the unordered CR array are ordered using the following:

(3) $$BC_{i}=\sum_{i=0}^{N}arg minCR_{i}$$

The best channel ($BC_{i}$) array is an ordered list of ranked channels with the lowest values (best ranked) first. This is useful for the algorithm since it uses the first ranked channel seeking available timeslots. If that particular channel is full, it keeps searching on the next ranked channel, and so on. The channel and timeslot selection process is described in Algorithm 2.
**Algorithm 2** Timeslot/channel selection algorithm.    **Input** The number of requiredCells    **Output** A cellList to schedule    **while**
selectedCells<requiredCells
**do**     Select a random timeslot     Select best ranked channel from the ordered list BC     Link← link with [timeslot,channel] from the slotframe     **if**
Link is available **then**      Append Link to cellList      selectedCells++     **end if**    **end while**

**Figure 3:**
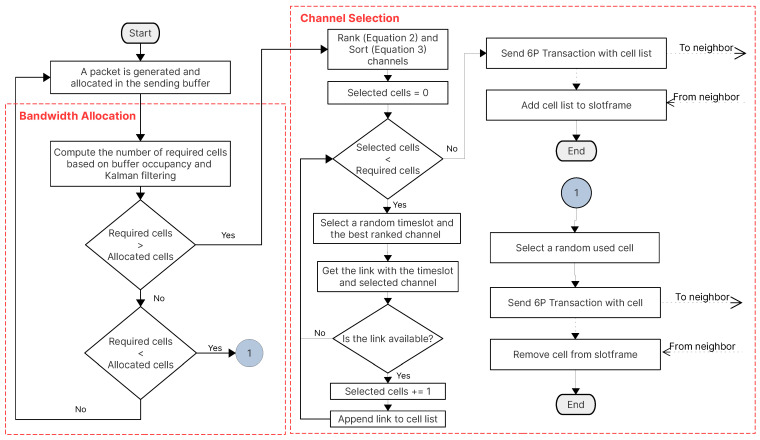
The complete bandwidth allocation, channel selection, and 6P negotiation processes of the Channel Ranking Scheduling Function (CRSF).

Depending on the number of required cells (computed with the Kalman filter), each node selects a random timeslot and the best-ranked channel to determine if the link is free or available. If so, the node appends the cell into a cell list to send it to its parent through a 6P Transaction. The parent must respond with a 6P Responseindicating the free links/cells it has. Upon receiving the message, the requesting node adds the cell list to its own schedule and sends another 6P Response to the parent to also add the cell list to its schedule. The CRSF is mainly comprised of two processes: the bandwidth allocation, in which a number of required cells based on the current occupancy of the sending buffer is computed; and the channel selection, in which the mathematical model described in Equations (2) and (3) is employed to generate a list of best channels based on the PDR, RSSI, and BN. The CRSF ends when the negotiation to add or remove cells with its neighbor node is achieved. Figure 3 shows the interaction of the processes of the CRSF.

## 5. Simulation Setup

Recent studies on distributed and autonomous scheduling functions [44] and KPIs [45] for 6TiSCH networks propose similar experimental setups to properly measure the performance of such functions. Our simulation setup follows the recommendations described in the literature in order to avoid biased setups [44,45]. This section describes the methodology for performance evaluation, the KPIs measured, and the simulated scenarios.

To compare the performance between different SFs deployed in a 6TiSCH network, we consider the following KPIs:

- PDR, defined as the ratio between the overall number of data packets sent and the number of packets received by the root or sink node. Such a metric measures the end-to-end reliability of the network.
- End-to-end latency, defined as the time interval of packet generation in the source node and the instant it is received at the final destination. This metric provides an insight into the performance of the bandwidth allocation mechanism.
- RDC, defined as the time a node is awake to send and receive data packets based on the number of scheduled cells in the slotframe. This metric defines how well the SF behaves when negotiating the allocation and deallocation of cells.
- First data packet delivery, defined as the instant the first data packet is received by the root node. This metric depicts the speed of the installation of the SF.

We implemented the CRSF in the Contiki-NG Operating System (OS), which is a popular OS for sensor networks with built-in support for 6TiSCH networks. We also used the Cooja network simulator, which is part of the tools available in Contiki-NG. The simulator supports the emulation of several platforms, allowing seamless deployments between simulated and real-world environments. In all our experimental scenarios, we used the RPL Lite routing protocol with default parameters (non-storing mode, no multiple instances of DODAG, and using the Minimum Rank with Hysteresis Objective Function (MRHOF) [46]) for multihop communication capabilities within the network.

**Figure 4:**
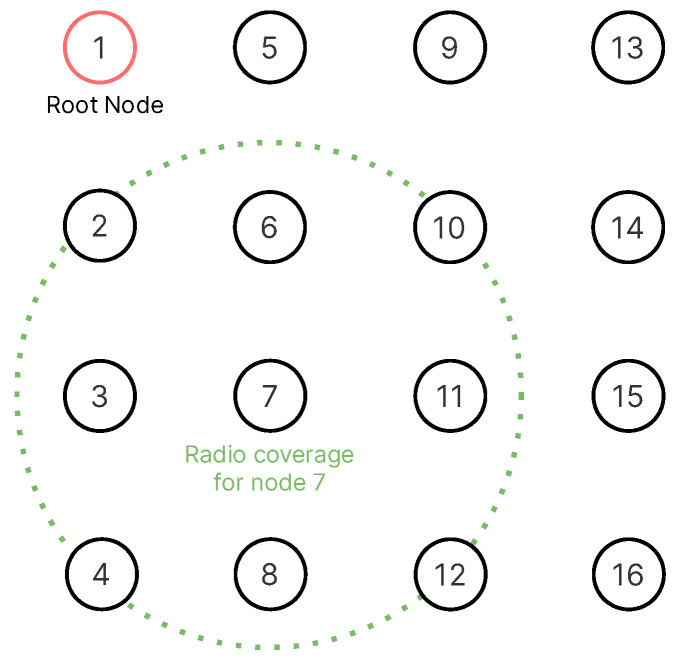
Example of a 4 × 4 grid topology. Each node is approximately 40 meters apart from the others. We assume there are no objects in between nodes.

The network topology used in the different scenarios is depicted in Figure 4. We used grid-based and uniformly distributed scenarios since this has been proposed in previous work for use as a reference model [44] to measure the SF performance and because this allows different RPL formations using the same physical network topology. We ran tests on network topologies with 16, 25, 49, and 64 nodes in 4×4, 5×5, 7×7, and 8×8 grids, respectively; and a 40 meter node separation from each other. By increasing the number of nodes in the network, we are targeting at measuring how well the SF scales. We used a 10 ms timeslot and slotframe lengths values of 17, 29, 47, and 101 slots for each network topology, respectively. The slotframe length was used as a parameter because shorter slotframes result in fewer cells to schedule. Thus, it has a direct effect on performance. We also used periodic and burst traffic patterns. This results in 32 different scenarios for each SF evaluated. The figure also depicts the approximate radio coverage for each node (50 meters according to the Unit Disk Graph Medium (UDGM) distance loss model from Cooja) to provide an estimate of the average number of hops in each topology.

The proposed CRSF is intended for industrial networks. In such scenarios, monitoring applications usually employ periodic upstream traffic with occasional traffic bursts from a limited number of nodes [45]. We target two traffic patterns, namely periodic and burst traffic patterns. In periodic traffic patterns, each node generates a packet every five seconds with a packet length of 60 bytes with the root node as the destination. In burst traffic patterns, each node generates 20 packets with a packet length of 60 bytes in uniform varying periods between two and 20 min. Such a configuration avoids flooding the network as it is expected that only a few nodes generate burst packets within a time period.

We compared the performance of CRSF to two popular SFs, namely the Minimal Scheduling Function (MSF) and Orchestra. Both SFs were chosen because they are already implemented in the Contiki-NG OS. The different test scenarios defined earlier were executed to collect data in the following manner: Each scenario ran for a fixed time of one hour. To obtain significant statistical results, we executed each experimental scenario 10 times and collected the data. We report the average values for each KPI. Traffic generation starts as soon as the root node is reachable on each node. Table 2 shows a summary of all parameter settings and traffic patterns used in our experiments.

## 6. Results Analysis

This section provides an analysis of our findings when measuring the performance of three different schedulers, namely our proposed CRSF, Orchestra, and the MSF, in the scenarios described earlier in Section 5.

### 6.1. Periodic Traffic Pattern

**Figure 5:**
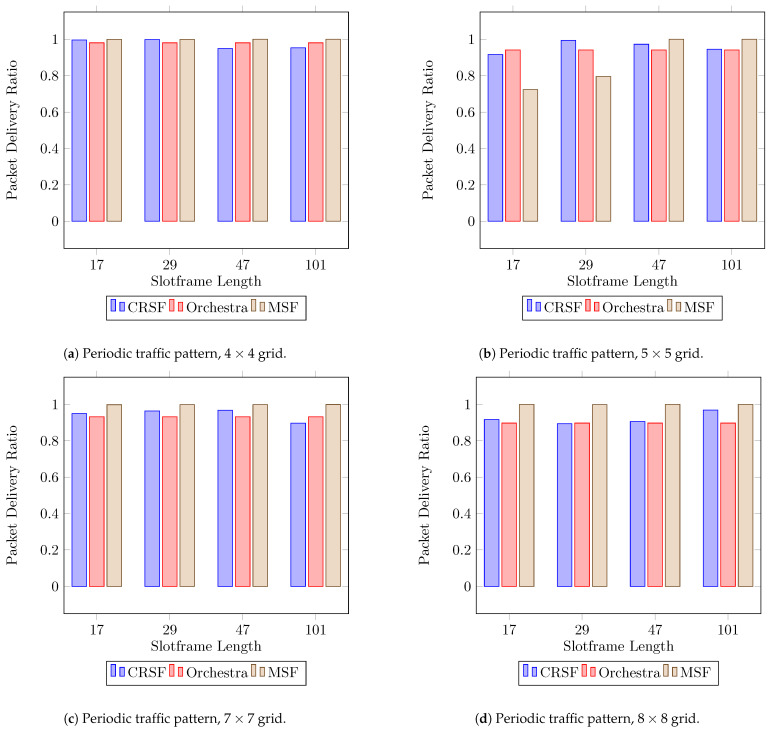
Average PDR performance using a periodic traffic pattern. Higher is better.

In periodic traffic patterns, each node in the network generates a 60 byte packet every five seconds with the root node as the destination. Based on the KPIs previously discussed, the PDR is probably the most important parameter since it expresses how reliable a particular SF is based on different setups. Figure 5 shows the results for the PDR when using the CRSF, Orchestra, and MSF, respectively, in 16 different scenarios where the topology and slotframe lengths are studied.

The CRSF, Orchestra, and MSF all deliver good performance on the PDR when the number of nodes is up to 7×7 (Figure 5c). However, in the case of the MSF, the indicator shows poor performance in a 5×5 topology and slotframe lengths of 17 and 29 timeslots (Figure 5b). Orchestra is slotframe length-independent; hence, its schedule is solely based on the number of nodes. On the other hand, the CRSF outperforms Orchestra in scenarios with 7×7 (Figure 5c) and 8×8 (Figure 5d) nodes and particularly with a slotframe length of 101, achieving up to a 0.968 PDR (see Table A1 in Appendix A for the supplementary data). This is important since industrial environments are expected to have dense node deployments.

**Figure 6:**
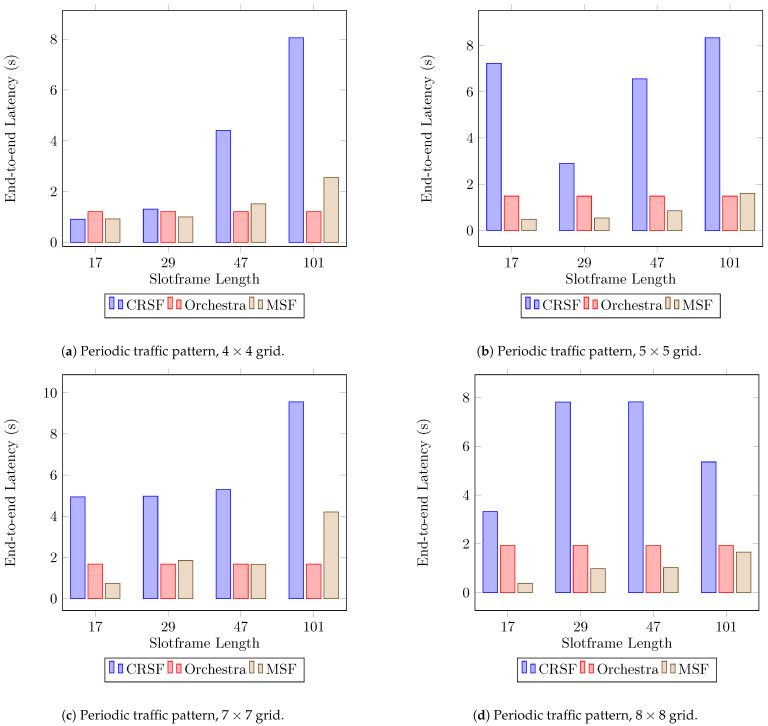
Average end-to-end latency performance using a periodic traffic pattern. Lower is better.

Figure 6 shows the results for end-to-end latency, measured as an average of the time it requires for the packets to travel from each node to the sink (see Table A2 in Appendix A for the supplementary data). Results show that the CRSF introduces significant delays in delivering packets to the sink (Figure 6b,d) since the bandwidth allocation algorithm is called whenever a packet is in the sending buffer. This also happens when packets are being routed from child nodes. However, this behavior can be seen as a compensation for the rapid adaptation to changes in the physical environment since the medium is verified each time a packet is to be sent. MSF, on the other hand, allocates negotiated cells every NUM_MAX_CELLS and uses a threshold to determine if the node is to schedule more cells or to delete scheduled cells. Orchestra defines its schedule just as the node is activated, assigning one cell of a certain channel to each node (being a neighbor or not); therefore, it does not base its schedule on the number of packets, but as a predefined rule.

The high rate calls of the CRSF to allow quick dynamic adaptations in bandwidth requirements present an impact on latency performance. Future research directions on 6TISCH SFs could focus on the trade-offs between the rate at which different SFs are called and the impact on KPIs such as latency and the RDC.

**Figure 7:**
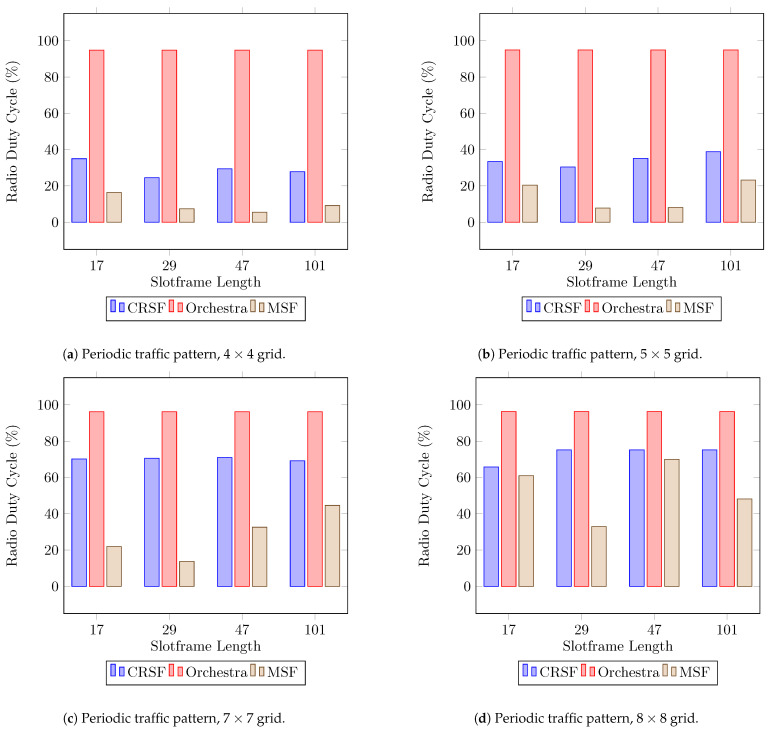
Average radio duty cycle performance using a periodic traffic pattern. Lower is better.

The RDC is a reflection of the amount of cells scheduled within the network. It also provides an indirect measurement for the energy consumption of the node [44]. Figure 7 shows the experimental results for this indicator measured as an average from the start of the network (see Table A3 in Appendix A for the supplementary data). The CRSF stays under 40% when the 4×4 and 5×5 topologies are used (Figure 7a,b), but scales up to 75.16% when the 8×8 grid topology is deployed (Figure 7d). Orchestra manages its schedule per node. As mentioned before, each node selects a channel offset and assigns one slot based on the node’s ID as *Tx*, *Rx*, and *Shared* options for data transmission and assigns all other slots on that channel to a broadcast address. Therefore, the RDC in Orchestra goes up to 96.34% in scenarios with 8×8 deployments, being above 94% for the RDC in all scenarios. On the other hand, the MSF shows low RDC measurements in scenarios with 4×4 and 5×5 nodes, which can be explained by the effective allocation algorithm since it also achieves higher throughput than the CRSF and Orchestra. The MSF shows optimal RDC measurements in scenarios with a low number of nodes (4×4 and 5×5 topologies). The MSF, however, starts to show a noticeable increase in the RDC when allocating cells in configurations with 7×7 and 8×8 grids (Figure 7c,d) as the slotframe length.

**Figure 8:**
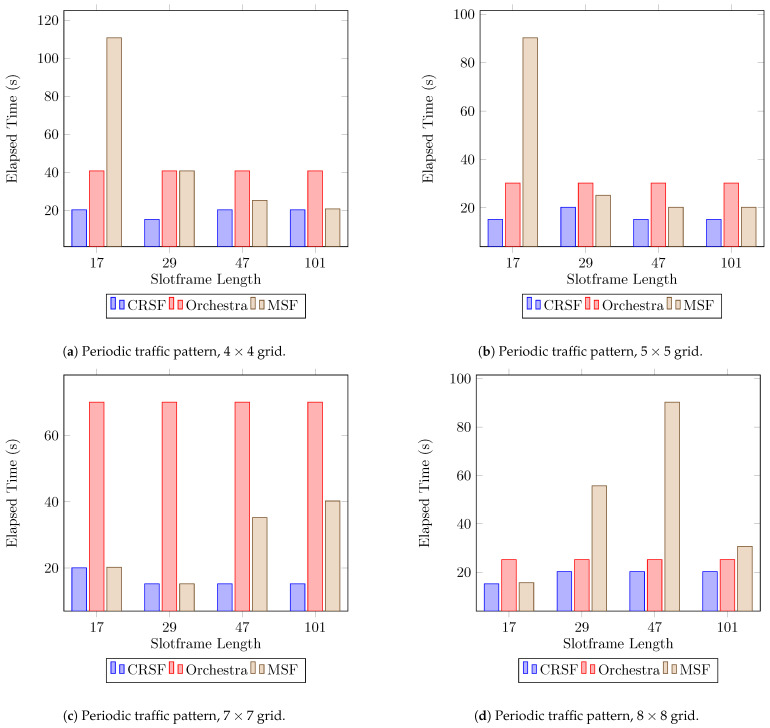
Results for the time elapsed for the first packet to be scheduled using a periodic traffic pattern. Lower is better.

The last performance indicator we included in our analysis is what we call first packet arrival, which measures the elapsed time from the start of the network until the first packet from any node arrives at the sink or root node. Figure 8 shows that the CRSF consistently outperforms Orchestra and the MSF across all scenarios. For scenarios with 7×7 nodes (Figure 8c), Orchestra exhibits poor performance. The MSF, however, shows the worst performance in scenarios with 4×4 (Figure 8a), 5×5 (Figure 8b), and 8×8 (Figure 8d) deployments (see Table A4 in Appendix A for the supplementary data). This indicator is important since it shows how fast a node is ready for transmission once the RPL knows how to reach the node.

### 6.2. Burst Traffic Pattern

Burst traffic patterns are present in industrial monitoring scenarios where large quantities of data are transmitted at irregular time intervals, for example when using vibration monitors. According to [45], burst sensors account for 10% of the logical roles in industrial environments, while the other 90% are periodic sensors such as temperature and pressure, among others. In our simulations, nodes generate burst traffic patterns of 20 packets with a packet length of 60 bytes in uniform varying periods between two and 20 min.

**Figure 9:**
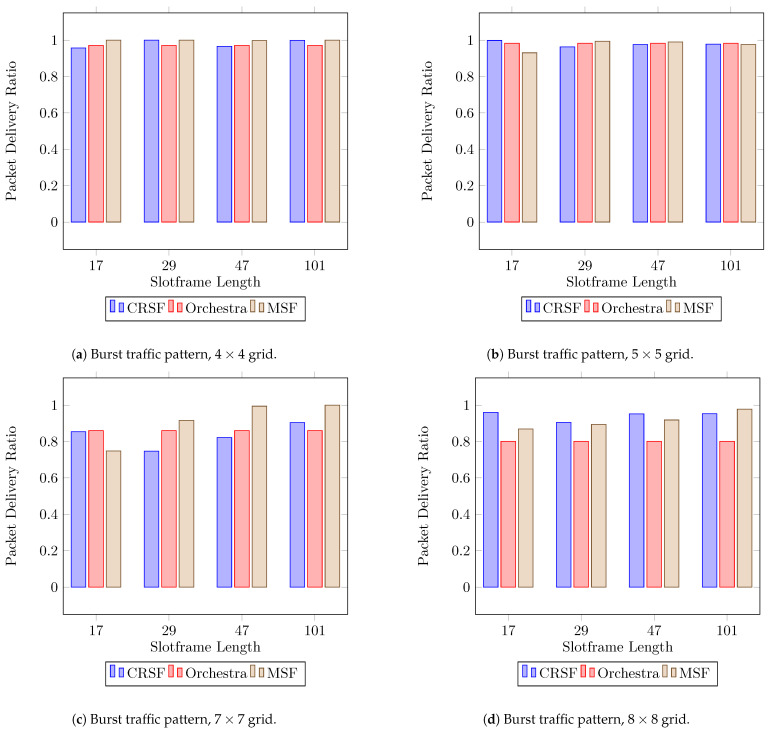
Average PDR performance using a burst traffic pattern. Higher is better.

Figure 9 shows the performance for the PDR for each SF studied (see Table A5 in Appendix A for the supplementary data). All three SFs, namely the CRSF, Orchestra, and MSF, show similar behaviors when using 4×4, 5×5, and 7×7 deployments (Figure 9a–c, respectively). for Orchestra and the MSF, however, the PDR tends to fall when 8×8 deployments are used (Figure 9d), whilst the CRSF achieves up to a 0.959 PDR in the same scenarios. As we discussed earlier in Section 6.1, the CRSF schedules packets based on the current buffer load; hence, it is demonstrated to be particularly effective and well-suited for scenarios where burst traffic is being generated.

**Figure 10:**
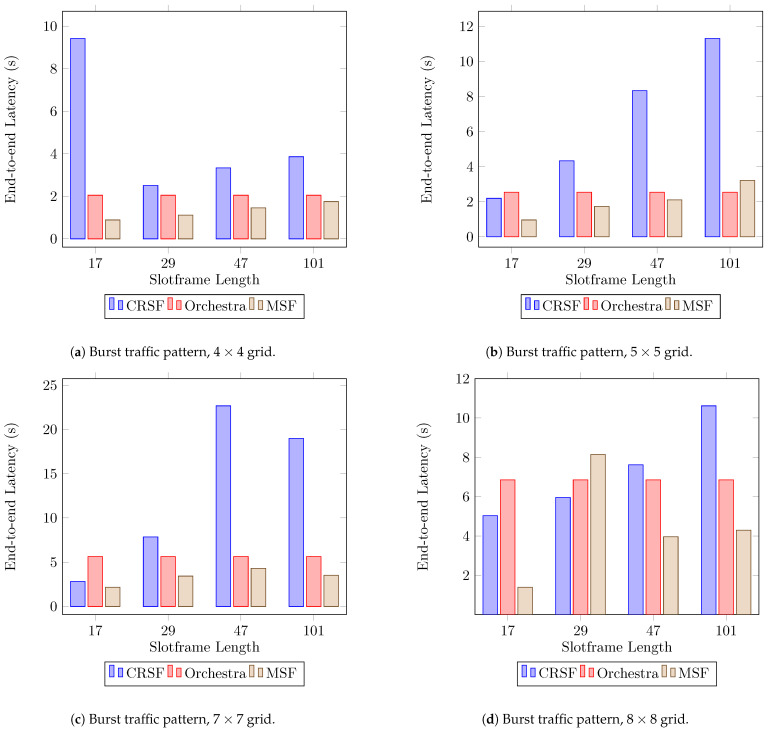
Average end-to-end latency performance using a burst traffic pattern. Lower is better.

The high PDR achieved by the CRSF in burst traffic scenarios is accompanied by larger latency compared to Orchestra and the MSF, as can be seen in Figure 10a,b (see Table A6 in Appendix A for the supplementary data). The CRSF shows a particularly large end-to-end latency in a 7×7 topology with slotframe lengths of 47 and 101 (Figure 10c). This, however, is not always true for larger node deployments since the CRSF behaves better in 8×8 deployments (Figure 10d).

**Figure 11:**
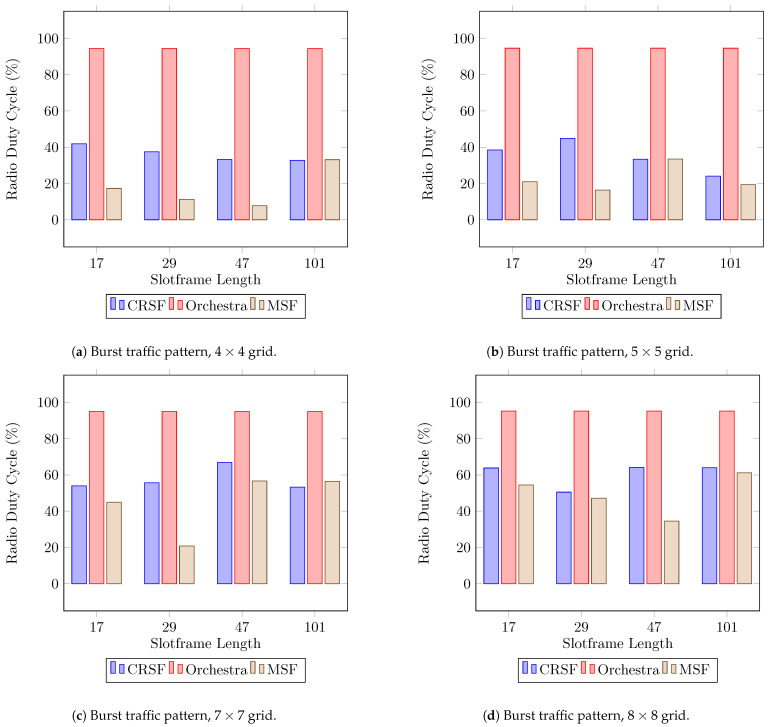
Average radio duty cycle performance using a burst traffic pattern. Lower is better.

Finally, the RDC indicator for burst traffic patterns is shown in Figure 11, where the CRSF stays under 40% in deployments of 4×4 (Figure 11a) and 5×5 (Figure 11b) and up to 63.82% in 7×7 (Figure 11c) and 8×8 (Figure 11d). The CRSF outperforms Orchestra in all scenarios by a large margin (see Table A7 in Appendix A for the supplementary data). The CRSF shows a similar performance when compared to the MSF as the number of nodes becomes larger. This behavior is similar to the measured results obtained in tests with periodic traffic patterns.

We did not include the results for our first packet arrival indicator since in scenarios with burst traffic patterns, the nodes are programmed to send their packets at random intervals. This restriction makes such an indicator irrelevant as a performance metric for such traffic patterns.

## 7. Conclusions

In this article, we define a new distributed SF called the Channel Ranking Scheduling Function (CRSF) for 6TiSCH networks. The SF is composed of a buffer-based bandwidth allocation algorithm based on the Kalman filter and a channel selection algorithm that incorporates several metrics such as the PDR, RSSI, and BN with an EWMA mechanism in order to select the best channel available. We also perform a detailed performance evaluation of three different scheduling functions for 6TiSCH networks, namely the CRSF, Orchestra, and MSF. We evaluate such SFs using network topologies of 4×4, 5×5, 7×7, and 8×8 grids to simulate high interference and usage for industrial networks. We also configure the slotframe length parameter to study its influence on the packet delivery ratio, end-to-end latency, and radio duty cycle.

Our results show that the CRSF effectively builds schedules based on the current characteristics of the network, achieving up to a 0.998 PDR in scenarios with 4×4 deployments, periodic traffic patterns, and a slotframe length of 29 and up to a 1.0 PDR in scenarios with 4×4 deployments, burst traffic patterns, and a slotframe length of 29. Our proposed SF, however, tends to have higher end-to-end latency compared to Orchestra and the MSF across all scenarios. This may be caused by the fact that the bandwidth allocation algorithm is executed each time a node is to send a packet, whether it is locally generated or a forwarded packet. The results for the radio duty cycle (RDC) indicator show acceptable performance for the CRSF with values ranging from 24.12% in scenarios with burst traffic, a slotframe length of 101 timeslots, and a 5×5 topology and up to 75.22% in scenarios with periodic traffic, a slotframe length of 47 timeslots, and an 8×8 topology, whilst Orchestra has the worst performance with values ranging from 94.52% in scenarios with burst traffic and a 4×4 topology (Orchestra is slotframe length-independent) and up to 96.34% in scenarios with periodic traffic and an 8×8 topology. The MSF, on the other hand, achieves the best RDC performance with values ranging from 5.5% in scenarios with periodic traffic, slotframe lengths of 47 timeslots, and a 4×4 topology and up to 70.0% in scenarios with periodic traffic, slotframe lengths of 47 timeslots, and an 8×8 topology.

From our results analysis, we can state that there is no SF suitable for every scenario and configuration. The CRSF is recommended to be used in scenarios with dense node deployment and where different sources of interference from other wireless networks such as Wi-Fi and environmental/background noise generated from heavy machinery are present since our ranking function incorporates the current characteristics of the physical medium for channel selection. The overall intention of defining a robust SF for 6TiSCH industrial networks is to enhance industrial logistic processes for either resource planning, warehouse management, transportation management, intelligent transportation, etc., because better and more efficient processes may result in lesser costs of operation and environmental pollution. Consistent with [31,45], we believe it is desirable to test different SFs, since they are an important part of any 6TiSCH network, in common, well-defined scenarios, and to avoid biased configurations to show the performance of the SFs [44]. Moreover, multiple SFs are expected to be used jointly to provide support for applications with different requirements.

Future work will focus on providing different metrics and weights to such metrics for the bandwidth allocation algorithm in order to support a wider range of 6TiSCH scenarios other than industrial environments. Furthermore, we want to study the impact of using different time intervals for the bandwidth allocation to take place. In our current implementation, the algorithm is executed each time a packet is to be sent on each node, which may incur a higher average end-to-end latency. We will also focus on lowering the RDC by adjusting the number of deleted cells, since our current implementation removes one cell each time the bandwidth allocation algorithm determines the current load is lower than the current number of allocated cells. This process can be further improved by removing a set of cells. This should improve the RDC of the CRSF since there will be fewer allocated cells.

## Figures and Tables

**Table 1:** Related work comparison. SF0, Scheduling Function Zero; MSF, Minimal Scheduling Function; BN, Background Noise.

Related Work	Scheduling Type	Bandwidth Estimation	Channel Selection
Palattella et al. [19]	Centralized	Based on network topology and buffer load	Sequential
Farías and Dujovne [20]	Centralized	Based on network topology	Sequential
SF0 [21]	Distributed	Based on buffer load	Based on PDR
Domingo-Prieto et al. [23]	Distributed	Based on PID of buffer load	Random
Muraoka et al. [24]	Distributed	Based on buffer load	Random
Accettura et al. [25]	Distributed	Based on network topology and buffer load	Sequential
Aijaz and Raza [26]	Distributed	Based on buffer load	Sequential
Chang et al. [27]	Distributed	Based on buffer load	Random
Karaagac et al. [28]	Hybrid	Based on QoS requirements	Based on PDR
Palattella et al. [30]	Distributed	Based on buffer load	Random
Righetti et al. [31]	Distributed	Based on buffer load and ETX	Random
Duquennoy et al. [32]	Distributed	Based on network topology	Sequential
MSF [34]	Distributed	Based on buffer load	Random
Hamza and Kaddoum [36]	Distributed	Prediction based on Poisson distribution	Random
Li et al. [38]	TSCH enhancement	N/A	Based on PDR
Gomes et al. [39]	TSCH enhancement	N/A	Based on PDR
Du and Roussos [40]	TSCH enhancement	N/A	Based on RSSI and BN
Tavakoli et al. [41]	TSCH enhancement	N/A	Based on RSSI and BN
Elsts et al. [42]	TSCH enhancement	N/A	Based on RSSI and PDR
Tavakoli et al. [43]	TSCH enhancement	N/A	Based on RSSI and BN
Our work	Distributed	Based on Kalman filtering of buffer load	Based on the PDR, RSSI, and BN

**Table 2:** Simulation parameter settings.

Parameter	Value
Number of nodes	16, 25, 49, 64 nodes.
Topology	4×4, 5×5, 7×7, 8×8
Timeslot duration	10 ms.
Slotframe length	17, 29, 47, 101 slots.
Simulation time	1 h per run.
Simulations per scenario	10 runs.
Periodic pattern	1 packet, 60 bytes length every 5 s.
Burst pattern	20 packets, 60 bytes each, every 2 to 20 min.

## Data Availability

Not applicable.

## Appendix A. Supplementary Data

**Table A1:** Results for the PDR with a periodic traffic pattern.

Size	Slotframe	CRSF	Orchestra	MSF
4×4	17	0.99659	0.98086	0.99977
	29	0.99836	0.98086	0.99919
	47	0.94903	0.98086	1.0
	101	0.95321	0.98086	1.0
5×5	17	0.91676	0.94118	0.72387
	29	0.99446	0.94118	0.79546
	47	0.97296	0.94118	1.0
	101	0.94565	0.94118	1.0
7×7	17	0.95006	0.93194	0.99839
	29	0.96423	0.93194	0.99921
	47	0.96792	0.93194	0.99910
	101	0.89663	0.93194	0.99922
8×8	17	0.91727	0.89744	0.99981
	29	0.89377	0.89744	0.99870
	47	0.90583	0.89744	1.0
	101	0.96883	0.89744	1.0

**Table A2:** Results for end-to-end latency (in seconds) with a periodic traffic pattern.

Size	Slotframe	CRSF	Orchestra	MSF
4×4	17	0.90 s	1.20 s	0.92 s
	29	1.30 s	1.20 s	0.99 s
	47	4.40 s	1.20 s	1.51 s
	101	8.05 s	1.20 s	2.55 s
5×5	17	7.22 s	1.48 s	0.48 s
	29	2.89 s	1.48 s	0.54 s
	47	6.55 s	1.48 s	0.85 s
	101	8.33 s	1.48 s	1.60 s
7×7	17	4.94 s	1.67 s	0.74 s
	29	4.97 s	1.67 s	1.84 s
	47	5.29 s	1.67 s	1.66 s
	101	9.55 s	1.67 s	4.20 s
8×8	17	3.32 s	1.92 s	0.38 s
	29	7.81 s	1.92 s	0.98 s
	47	7.82 s	1.92 s	1.02 s
	101	5.35 s	1.92 s	1.65 s

**Table A3:** Results for the Radio Duty Cycle (RDC) with a periodic traffic pattern.

Size	Slotframe	CRSF	Orchestra	MSF
4×4	17	34.97%	94.82%	16.40%
	29	24.49%	94.82%	7.38%
	47	29.42%	94.82%	5.50%
	101	27.84%	94.82%	9.14%
5×5	17	33.44%	94.92%	20.4%
	29	30.44%	94.92%	7.76%
	47	35.09%	94.92%	8.0%
	101	38.86%	94.92%	23.24%
7×7	17	70.15%	96.19%	21.92%
	29	70.59%	96.19%	13.66%
	47	71.00%	96.19%	32.57%
	101	69.15%	96.19%	44.59%
8×8	17	65.75%	96.34%	60.96%
	29	75.18%	96.34%	32.95%
	47	75.22%	96.34%	70.0%
	101	75.16%	96.34%	48.17%

**Table A4:** Results for first packet reception (in seconds) with a periodic traffic pattern.

Size	Slotframe	CRSF	Orchestra	MSF
4×4	17	20.24 s	40.72 s	110.72 s
	29	15.24 s	40.72 s	40.72 s
	47	20.24 s	40.72 s	25.24 s
	101	20.24 s	40.72 s	20.72 s
5×5	17	15.25 s	30.25 s	90.25 s
	29	20.24 s	30.25 s	25.24 s
	47	15.25 s	30.25 s	20.25 s
	101	15.25 s	30.25 s	20.25 s
7×7	17	20.07 s	70.07 s	20.24 s
	29	15.24 s	70.07 s	15.24 s
	47	15.24 s	70.07 s	35.24 s
	101	15.24 s	70.07 s	40.24 s
8×8	17	15.24 s	25.24 s	15.71 s
	29	20.24 s	25.24 s	55.71 s
	47	20.24 s	25.24 s	90.24 s
	101	20.24 s	25.24 s	30.71 s

**Table A5:** Results for the PDR with a burst traffic pattern.

Size	Slotframe	CRSF	Orchestra	MSF
4×4	17	0.95701	0.970833	1.0
	29	1.0	0.970833	1.0
	47	0.96551	0.970833	0.99823
	101	0.99910	0.970833	1.0
5×5	17	0.99838	0.983	0.93039
	29	0.96325	0.983	0.99361
	47	0.97560	0.983	0.98989
	101	0.97761	0.983	0.97617
7×7	17	0.85406	0.85994	0.74797
	29	0.74710	0.85994	0.91632
	47	0.82244	0.85994	0.99457
	101	0.90379	0.85994	1.0
8×8	17	0.95979	0.80147	0.86886
	29	0.90466	0.80147	0.89413
	47	0.95220	0.80147	0.91858
	101	0.95263	0.80147	0.97797

**Table A6:** Results for end-to-end latency (in seconds) with a burst traffic pattern.

Size	Slotframe	CRSF	Orchestra	MSF
4×4	17	9.42 s	2.05 s	0.88 s
	29	2.51 s	2.05 s	1.11 s
	47	3.33 s	2.05 s	1.45 s
	101	3.86 s	2.05 s	1.75 s
5×5	17	2.19 s	2.53 s	0.95 s
	29	4.32 s	2.53 s	1.73 s
	47	8.32 s	2.53 s	2.10 s
	101	11.30 s	2.53 s	3.21 s
7×7	17	2.82 s	5.63 s	2.16 s
	29	7.85 s	5.63 s	3.42 s
	47	22.66 s	5.63 s	4.29 s
	101	18.98 s	5.63 s	3.52 s
8×8	17	5.03 s	6.85 s	1.39 s
	29	5.95 s	6.85 s	8.14 s
	47	7.61 s	6.85 s	3.96 s
	101	10.61 s	6.85 s	4.29 s

**Table A7:** Results for the RDC with a burst traffic pattern.

Size	Slotframe	CRSF	Orchestra	MSF
4×4	17	41.88%	94.52%	17.33%
	29	37.53%	94.52%	11.28%
	47	33.28%	94.52%	7.76%
	101	32.77%	94.52%	33.15%
5×5	17	38.53%	94.64%	21.01%
	29	44.95%	94.64%	16.35%
	47	33.38%	94.64%	33.53%
	101	24.12%	94.64%	19.38%
7×7	17	53.96%	94.98%	44.97%
	29	55.64%	94.98%	20.81%
	47	66.91%	94.98%	56.64%
	101	53.29%	94.98%	56.46%
8×8	17	63.82%	95.20%	54.49%
	29	50.50%	95.20%	47.13%
	47	64.20%	95.20%	34.5%
	101	63.98%	95.20%	61.13%
